# Characterization of Antioxidant Activity of Heated Mycosporine-like Amino Acids from Red Alga Dulse *Palmaria palmata* in Japan

**DOI:** 10.3390/md20030184

**Published:** 2022-03-01

**Authors:** Yuki Nishida, Wataru Saburi, Yoshikatsu Miyabe, Hideki Kishimura, Yuya Kumagai

**Affiliations:** 1 Marine Chemical Resource Development, Graduate School of Fisheries Sciences, Hokkaido University, Hakodate 041-8611, Hokkaido, Japan; karakuchi@eis.hokudai.ac.jp (Y.N.); yoshikatsu_miyabe@aomori-itc.or.jp (Y.M.); 2 Fundamental AgriScience Research, Research Faculty of Agriculture, Hokkaido University, Kita 9 Nishi 9, Kita-ku, Sapporo 060-8589, Hokkaido, Japan; saburi@chem.agr.hokudai.ac.jp; 3 Aomori Prefectural Industrial Technology Research Center, Food Research Institute, 2-10 Chikkogai, Hachinohe-shi 031-0831, Aomori-ken, Japan; 4 Marine Chemical Resource Development, Faculty of Fisheries Sciences, Hokkaido University, Hakodate 041-8611, Hokkaido, Japan

**Keywords:** red alga dulse, mycosporine-like amino acids, palythine, antioxidant activity

## Abstract

We recently demonstrated the monthly variation and antioxidant activity of mycosporine-like amino acids (MAAs) from red alga dulse in Japan. The antioxidant activity of MAAs in acidic conditions was low compared to that in neutral and alkali conditions, but we found strong antioxidant activity from the heated crude MAA fraction in acidic conditions. In this study, we identified and characterized the key compounds involved in the antioxidant activity of this fraction. We first isolated two MAAs, palythine, and porphyra-334, from the fraction and evaluated the activities of the two MAAs when heated. MAAs possess absorption maxima at around 330 nm, while the heated MAAs lost this absorption. The heated MAAs showed a high ABTS radical scavenging activity at pH 5.8–8.0. We then determined the structure of heated palythine via ESI-MS and NMR analyses and speculated about the putative antioxidant mechanism. Finally, a suitable production condition of the heated compounds was determined at 120 °C for 30 min at pH 8.0. We revealed compounds from red algae with antioxidant activities at a wide range of pH values, and this information will be useful for the functional processing of food.

## 1. Introduction

Marine organisms, e.g., dinoflagellates, cyanobacteria, and macroalgae, synthesize and accumulate mycosporine-like amino acids (MAAs) as natural compounds to protect themselves from UV radiation damages [1,2]. MAAs are secondary metabolites of low molecular weight (<400 Da), with maximum absorption values ranging from 310 to 360 nm and high molar extinction coefficients (28,100 to 50,000 M^−1^ cm^−1^) [3,4]. It was previously reported that 97% of the light energy absorbed by shinorine and porphyra-334 is converted into heat [5]. In addition, these compounds hardly photodegrade and show high stability. In cyanobacteria and red algae, MAAs also have a role as nitrogen storage substances. It is thought that MAAs are degraded in the cell to release nitrogen atoms when needed, but the mechanism is not yet understood [6]. To date, no toxicity of MAAs from red algae or cyanobacteria to human cells has been reported [4,7]. Many functions of MAAs have been reported, including as sunscreens [4], anticancer agents for melanoma cells [7], activators of fibroblasts cell proliferation [1], antiphotoaging compounds [8], and antioxidants [9,10].

We previously studied dulse (*Palmaria palmata*) harvested in Usujiri, Hakodate, Japan [11] and revealed that it contains abundant protein [12]. The major protein component, phycoerythrin (PE), is a potential source of peptides for angiotensin I converting enzyme inhibition [13,14] and chromophores with antioxidant activity [15]. We then prepared xylooligosaccharides from xylan, a cell wall component of the dulse [16,17,18]. In addition, we recently found that MAAs are relatively abundant in Japanese dulse, and the monthly variation in their content was clarified [19,20]. We also revealed that two major MAAs from the dulse, palythine, and porphyra-334, show high activity in alkaline conditions and low activity in acidic conditions [20]. The foods we consume have various pH levels. Hence, it is important to understand the effect of pH on the antioxidant function of MAAs when using them as food ingredients for their antioxidant properties.

Furthermore, Yoshiki et al. (2009) reported that the heating of the compound porphyra-334, one of the major MAAs of red algae, produces strong antioxidant activity [21]. However, this antioxidant activity was shown under only one pH condition (2-morpholinoethanesulfonic acid buffer at pH 6.0). Therefore, in this study, we prepared two major MAAs (palythine and porphyra-334) from Japanese dulse [19] and investigated the effects of heating and pH on their antioxidant activities. In addition, the novel structure of the heated product of palythine was determined by ESI-MS and NMR analyses, and its antioxidant mechanism was deduced. Furthermore, we clarified the optimal condition for the preparation of heated MAAs. We believe that these results are useful information to enhance the functionality of red algae as food materials.

## 2. Results and Discussion

### 2.1. Antioxidant Activity of Heated Crude MAAs

**Figure 1:**
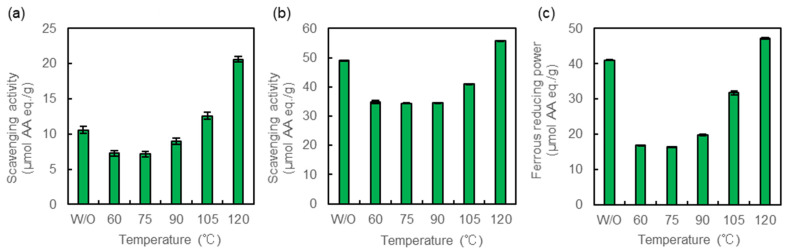
Antioxidant activity of crude MAAs. (**a**) DPPH radical scavenging activity, (**b**) ABTS radical scavenging activity, and (**c**) ferrous reducing power. Crude MAAs were heated at 60, 75, 90, 105, and 120 °C. Antioxidant activity is expressed as ascorbic acid equivalents (µmol AA eq./1 g sample). Three assays were performed at pH 7.4. Data are the mean values ± standard errors of three independent experiments. W/O: without heat treatment.

We first evaluated the antioxidant activity of the heated crude MAAs using DPPH and ABTS radical scavenging and ferrous reducing power assays (Figure 1). Antioxidant activities of crude MAAs treated at 60–105 °C were decreased in the three assays, except for DPPH radical scavenging at 105 °C. However, the activities increased in the sample treated at 120 °C. The DPPH radical scavenging activity was 2.0-fold higher in the treated sample than in the untreated sample (Figure 1a). The increases were also confirmed by the assays of ABTS radical scavenging and ferrous reducing power, i.e., 1.1- and 1.2-fold higher than that of the untreated sample (Figure 1b,c).

Many antioxidant active compounds have been identified from algae and plants, e.g., catalase [22], phycoerythrin [15], fucoidan [23], ascorbic acid [24], insoluble antioxidants [25,26], phlorotannins [27,28], catechins [29], scytonemin [30], and phenolic compounds (e.g., gentisic acid, protocatechuic acid, and gallic acid) [31]. These compounds have been shown to be unstable following heating processes, such as sun drying, storage, boiling, and roasting [32,33]. Gallic acid is especially unstable, with an initial thermal decomposition temperature of 68 °C [34]. Therefore, the decrease in the activity in the heated crude MAAs was due to the thermal decomposition of antioxidants. On the other hand, the activity of a sample from *Neopyropya yezoensis* containing MAAs was enhanced at 100–120 °C [21]. Therefore, we purified MAAs, prepared heated MAAs, and evaluated the samples’ antioxidant activities.

### 2.2. ABTS Radical Scavenging Activity of Heated MAAs

We purified palythine and porphyra-334 from crude MAAs and prepared heated palythine and heated porphyra-334. Their ABTS radical scavenging activities were measured at pH 5.8, 6.6, 7.4, and 8.0 (Table 1). The IC_50_ values of heated palythine were improved at the tested pHs. The activities at pH 7.4 and 8.0 were 2.7- and 1.8-fold higher than those of palythine, respectively. The IC_50_ values of heated porphyra-334 were also improved compared with that of porphyra-334, i.e., improvements 2.2-fold at pH 7.4 and 3.3-fold at pH 8.0. MAAs showed low antioxidant activity in acidic conditions (pH 5.8 and 6.6). The activities of heated MAAs were improved from those of untreated MAAs and superior to those of ascorbic acid.

### 2.3. Structural Changes of Heated MAAs

**Figure 2:**
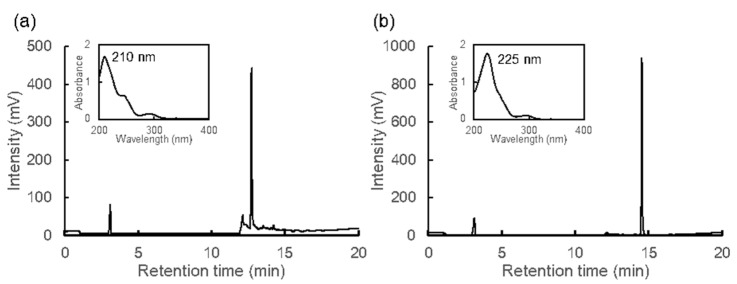
HPLC chromatograms and absorption spectra. (**a**) Heated palythine and (**b**) heated porphyra-334. Spectra in figures show the peaks from 12.72 min (**a**) and 14.53 min (**b**).

Although the antioxidant activities of MAAs decreased in acidic conditions, heated MAAs showed high activities at a wide range of pH (Table 1). Therefore, we expected structural changes in MAAs and evaluated them using HPLC, spectrum analysis, and ESI-MS. Palythine and porphyra-334 were eluted at the retention times of 4.79 and 7.73 min, respectively [14]. However, the heated samples lost their absorption at around 330 nm and were eluted at 12.72 min for heated palythine and 14.53 min for heated porphyra-334, with absorption maxima (λ_max_) of 210 and 225 nm, respectively (Figure 2).

**Figure 3:**
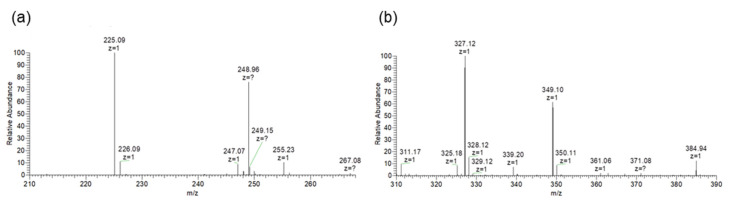
ESI-MS spectra. (**a**) Heated palythine. Peak of [M − H]^−^ at *m*/*z* 225.1 was obtained. (**b**) Heated porphyra-334. Peak [M − H]^−^ at *m*/*z* 327.1 was obtained.

We then purified the heated compounds via HPLC and subjected them to ESI-MS analysis (Figure S1). Heated palythine and heated porphyra-334 showed the prominent ion peaks of deprotonated molecules ([M − H]^−^) at *m*/*z* 225.1 and 327.1, respectively (Figure 3). Comparing molecular weight between the MAAs and heated MAAs, heated MAAs lost 18 Da, which corresponded to the molecular weight of water. The *m*/*z* of heated porphyra-334 corresponded to the previously reported Nori antioxidant compound (NAC) [21]. Therefore, we focused on the heated palythine, which has not previously been described, and attempted to determine its structure.

### 2.4. NMR Analyses of Heated Palythine

**Figure 4:**
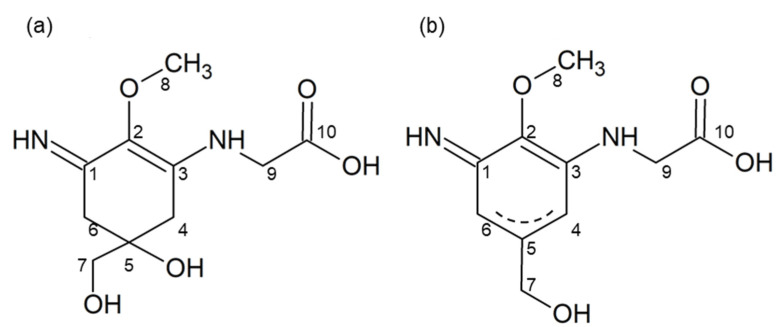
Estimated chemical structure of heated palythine. (**a**) Palythine and (**b**) heated palythine. Numbers in the figure relate to Table 2.

The ^1^H-NMR spectrum of heated palythine indicated a good correlation with the chemical shift of palythine [3], except for the chemical shifts of H-4 and H-6, which were shifted to lower magnetic fields (4.58–4.66 ppm) (Table 2, Figure S2). The ^13^C-NMR spectrum of heated palythine showed four peaks in the saturated carbon region (29.0, 47.4, 63.5, and 65.8 ppm), while palythine had six peaks (33.5, 35.7, 47.0, 58.9, 67.3, and 71.2 ppm) (Figure S3). Heated palythine showed six peaks in the unsaturated carbon region (127.0, 141.2, 144.4, 160.9, 165.9, and 178.2 ppm) and palythine showed four peaks (125.9, 158.7, 161.5, and 174.6 ppm). Therefore, the chemical structure of heated palythine indicated the formation of a double bond between C-4 and C-5 or between C-5 and C-6 (Figure 4). The dehydration pattern of palythine was consistent with that of porphyra-334 [24], implying that other imino-MAAs show a similar dehydration pattern.

### 2.5. Presumed Stabilization Mechanisms of Heated MAAs

In this study, heated MAAs showed antioxidant activity at a wide range of pH values compared with MAAs. The difference in the chemical structures is the formation of a double bond (Figure 4). Namely, MAAs are dehydrogenated at the methylene of C-4 and C-6; meanwhile, the heated MAAs were found to have a resonance-type double bond between C-4 and C-5 or between C-5 and C-6 (Figure 4b). We presumed these to be stabilization mechanisms based on the structural differences.

**Figure 5:**
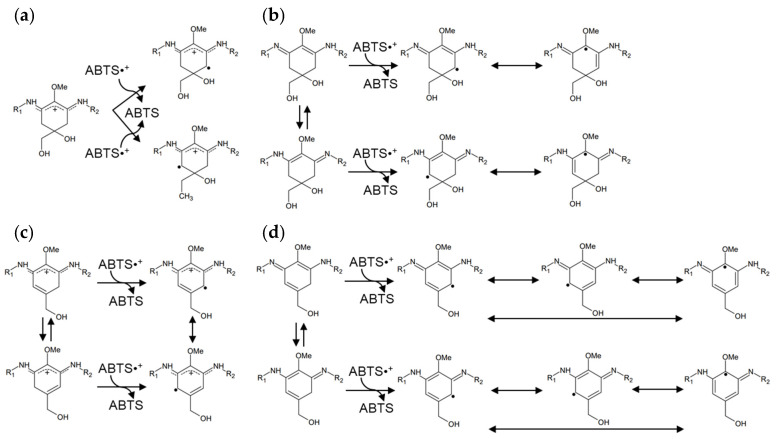
Presumed stabilization mechanisms of radical electrons in MAAs and heated MAAs. (**a**) MAAs in acidic conditions; (**b**) MAAs in alkali conditions; (**c**) heated MAAs in acidic conditions; and (**d**) heated MAAs in alkali conditions. Porphyra-334: R_1_ = CH(COOH)CHCH_3_OH; R_2_ = CH_3_COOH, Palythine: R_1_ = H; R_2_ = CH_3_COOH, Usujirene: R_1_ = CH=CHCH_3_; R_2_ = CH_3_COOH.

The activities of imino-MAAs are greatly affected by pH [35,36,37]. Namely, the delocalization mechanism of radical electrons in the cyclohexene group depends on the pH. The radical electron cannot widely delocalize in MAAs in acidic conditions, resulting in weak antioxidant activity (Figure 5a). On the other hand, the radical electrons of MAAs were already delocalized in alkali conditions (Figure 5b), showing stronger antioxidant activity than that in acidic conditions. Because of the double bonds in heated MAAs, radical electrons were delocalized in acidic and alkali conditions (Figure 5c,d). The delocalization of radical electrons in heated MAAs in the alkali condition increased, resulting in enhanced antioxidant activity (Table 1).

### 2.6. Efficient Production of Heated MAAs

**Figure 6:**
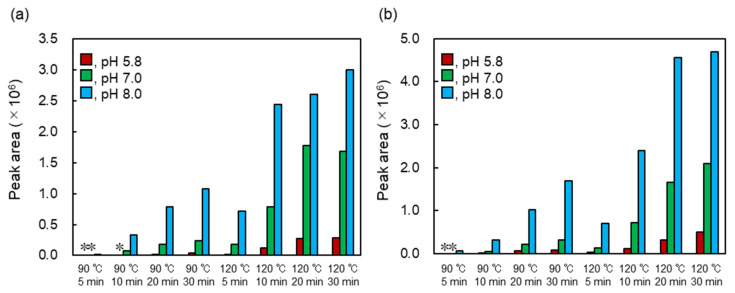
HPLC peak areas of heated MAAs. (**a**) Production of heated palythine and (**b**) production of heated porphyra-334. The detection wavelength was set at 220 nm. Asterisk means undetected.

Heated MAAs showed strong antioxidant activities with a wide range of pH values. The optimum production conditions of heated MAAs were determined by pH, heating time, and temperature. The amounts of heated MAAs were evaluated by the HPLC peak area (Figure 6). Conversion was well progressed at 120 °C compared to the samples at 90 °C. In addition, the reaction at pH 8.0 was suitable for the conversion. The residual MAAs after heating at 120 °C for 30 min (pH 8.0) were evaluated, showing that palythine and porphyra-334 remained at 0% and 4.3%, respectively. From these results, dehydration of MAAs was determined to progress at high temperatures and in alkali conditions.

As shown in Figure 2, the maximum absorption wavelength of MAAs shifted to the shorter wavelength side upon heat treatment. However, there is little information on any other physical properties and health functionalities of heat-treated MAAs. In the case of NAC, dried nori products contain little NAC, while roasted nori products contain 1–2% per dry weight, suggesting that the roasting process converts much of the porphyra-334 into NAC [21]. Because of the long history of eating roasted seaweed in Japan, NAC is considered a safe compound.

### 2.7. Character of Heated Usujirene

**Figure 7:**
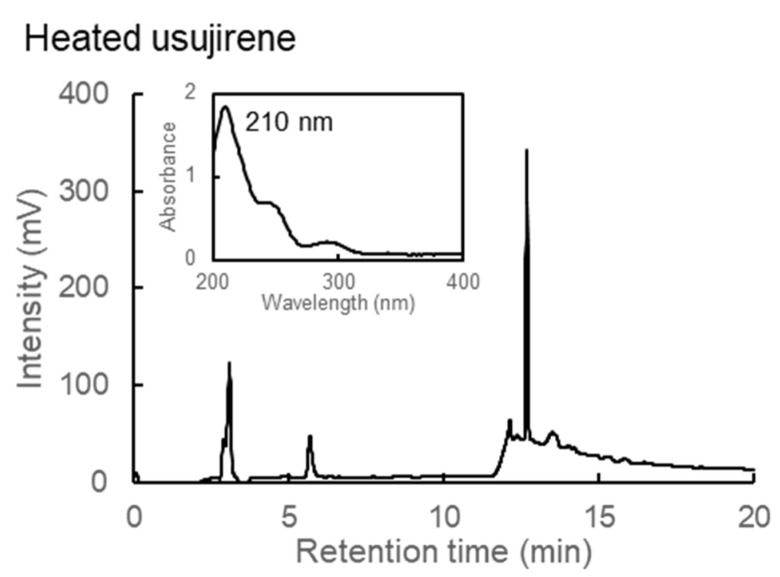
HPLC chromatograms and absorption spectra of heated usujirene.

Usujirene is one of the MAAs found in dulse (*P. palmata*) from Usujiri, Hakodate, Japan. Usujirene is easily hydrolyzed and converted into palythine [38,39,40,41]. We predicted that heated usujirene would be eventually converted into heated palythine. Therefore, we purified usujirene and evaluated the heated product. Usujirene was eluted at 13.36 min with λ_max_ at 357 nm [19], while heated usujirene lost absorption at 357 nm. Instead, peak elution at 12.69 min with a λ_max_ at 210 nm was obtained (Figure 7). The peak pattern corresponded to heated palythine, suggesting that the usujirene was converted into palythine and then dehydrated into heated palythine.

Dehydration by heating does not occur in all MAAs. For example, mycosporine-glycine, classified as an oxo-MAA, is converted into glycine and 6-deoxygadusol [38,39]. Further, 6-Deoxygadusol is also a strong antioxidant compound, indicating that the heating processes of algae increase antioxidant compounds.

## 3. Materials and Methods

### 3.1. Algal Material

Dulse samples (*P. palmata*) were collected in Usujiri, Hakodate, Japan (41°56′ N, 40°56′ E) at a depth of 1 m from the surface of the sea on 25 February 2019. Dulse was washed thoroughly with tap water and lyophilized in a dry chamber using a freeze dryer (FD-1, EYLA, Tokyo, Japan) under a vacuum of 65–70 Pa and a trapping temperature of −45 °C for 12 h. Dried dulse was powdered using a Wonder Blender WB-1 (Osaka Chemical Co., Osaka, Japan).

### 3.2. Preparation of Crude MAAs

Crude MAAs were prepared according to the method described by Nishida et al. (2020) [19]. Namely, the dulse powder was suspended in 20 volumes (*w*/*v*) of distilled water at 4 °C for 6 h. After centrifugation (27,200× *g*, 10 min), the supernatant was lyophilized. The extract was then resuspended in 20 volumes (*w*/*v*) of methanol at 4 °C for 2 h. After centrifugation (27,200× *g*, 15 min), the supernatant was evaporated and lyophilized. The dried sample was labeled as crude MAAs and used for the following analyses.

### 3.3. Preparation of Purified MAAs from Dulse

MAAs were purified from crude MAAs [19]. Namely, crude MAAs were dissolved in HPLC-grade water containing 0.1% trifluoroacetic acid (TFA) (*v*/*v*), followed by sequential filtration through 0.22 and 0.20 μm membrane filters. The filtrated MAA solution was subjected to HPLC with a Mightysil RP-18GP column (5 µm, 10 × 250 mm) (Kanto Kagaku, Tokyo, Japan) and eluted with an isocratic solvent of 0.1% TFA for 7 min and a linear gradient of acetonitrile (0–70%) containing 0.1% TFA for 13 min at a flow rate of 4.73 mL/min, with the column oven temperature at 40 °C. Elution was detected at 330 nm. The fractions (retention time of 4.42, 4.79, 7,73 and 13.36 min) were collected and evaporated to obtain purified palythine, porphyra-334, and usujirene.

### 3.4. Heat Treatment of MAAs

Crude MAAs were dissolved in 0.1 M phosphate buffer (pH 7.4) at a concentration of 5 mg/mL and heated at 60, 75, 90, 105, or 120 °C for 30 min with a dry bath (thermostat) (MD-02N, Major Science, Saratoga, CA, USA). The samples were cooled in a refrigerator to 4 °C and then kept at −30 °C until analyses.

Purified palythine and porphyra-334 were dissolved in 20 mM phosphate buffer (pH 7.4), and then the solutions were autoclaved (MLS-2420, SANYO Electric, Osaka, Japan) at 121 °C for 20 min.

### 3.5. Assay of Antioxidant Activity

Three methods of assessing antioxidant activity (DPPH and ABTS radical scavenging assay and ferrous reducing power assay) were employed. DPPH radical scavenging assay was performed according to the method of a previous study [7] with some modifications. First, 0.5 mL of sample or control (distilled water) was mixed with 0.5 mL of 0.2 M phosphate buffer (pH 7.4) and 0.75 mL ethanol containing 0.5 mM DPPH. The mixtures were reacted for 20 min at room temperature in the dark. Because a slight precipitate was produced after the reaction, the solutions were centrifuged at 4 °C, 2000× *g* for 10 min, and the absorbance at 517 nm was measured. Since MAAs are water-soluble compounds, we used ascorbic acid, which is also a water-soluble antioxidant. This was also because ascorbic acid is often used as a positive control in antioxidant research in the food and cosmetic fields.

The ABTS radical scavenging assay was performed according to the method of a previous study [19]. The ABTS·+ solution was prepared by mixing an equal volume of 14.8 mM ABTS and 5.2 mM potassium persulphate and incubating for 12 h at room temperature in the dark. The absorbance at 734 nm of ABTS·+ solution was adjusted to 1.00 ± 0.02 by adding 0.2 M phosphate buffer (pH 5.8, 6.6, 7.4, or 8.0). The assay was conducted as follows: 50 µL of sample or control (distilled water) was mixed with 950 µL of the ABTS·+ solution or the phosphate buffer. The mixture was then incubated for 2 h at room temperature in the dark. After incubation, the solution was centrifuged at 4 °C, 2000× *g* for 5 min, and the supernatant absorbance was measured at 734 nm. Ascorbic acid was used as a standard compound.

The ferrous reducing power assay was performed according to the method of a previous study [42]. First, 0.4 mL of sample or control (distilled water) was mixed with 0.4 mL of 0.2 M phosphate buffer (pH 7.4) and 0.4 mL of 1% potassium ferricyanide. The mixture was incubated for 20 min at 50 °C. Subsequently, 0.4 mL of 10% trichloroacetic acid was added to the mixture. Measures of 0.8 mL of samples were extracted and then mixed with 0.96 mL of 0.017% ferric chloride and incubated for 10 min at room temperature. The absorbance of solutions was measured at 700 nm using ascorbic acid as a standard.

### 3.6. Spectrophotometric Analysis of Heat-Treated MAAs

The thermal derivatives of MAA solutions were analyzed in the UV-visible ray absorption spectrum using a spectrophotometer (200–400 nm, UV-1800, Shimadzu, Kyoto, Japan).

### 3.7. ESI-MS Analysis of Heat-Treated MAAs

The mass-to-charge ratios of thermal derivatives of MAAs were determined via the electrospray ionization-ion trap mass spectrometry (ESI-MS) method using a Thermo Scientific Exactive (Thermo Scientific, Waltham, MA, USA). For ESI-MS analysis, the compounds were dissolved in an appropriate amount of ultrapure water. The detection was performed in the negative mode.

### 3.8. ^1^H- and ^13^C-NMR Analyses of Heat-Treated Palythine

The purified thermal derivative of palythine was analyzed and its structure confirmed through ^1^H- and ^13^C-NMR analysis in a Bruker AVANCE Neo (Bruker, MA, USA) (500.13 MHz for ^1^H-NMR and 125.77 MHz for ^13^C-NMR) at 300 K. For NMR analysis, the compound was dissolved in D_2_O (99.8% deuterium) at a concentration of 50 mg/mL, and TSP (trimethylsilyl propanoic acid) was used as an internal standard.

### 3.9. Efficient Production of Heated MAAs

Efficient production conditions of heated MAAs were determined for pH, heating time, and temperature. Palythine and porphyra-334 were dissolved in 0.1 M phosphate buffer (pH 5.8, 7.0, or 8.0) at the concentrations of 0.32 and 0.16 mM, respectively. The MAA-containing solution was heated at 90 or 120 °C for 5, 10, 20, or 30 min with a dry bath (thermostat) and then cooled in a refrigerator at 4 °C.

## 4. Conclusions

One of the functions of MAAs is antioxidant activity. However, the activity varies with the pH. We found that heated MAAs showed a high activity across a broad pH range. The structure of heated palythine was determined by ESI-MS and NMR, showing that an additional double bond occurred in the cyclohexene group. The putative delocalization mechanism of radical electrons was speculated to have produced the double bond that contributed to the stabilization of the antioxidant compounds. MAAs were effectively converted into heated MAAs under high temperatures and in alkali conditions. The information is useful for algae food processing using antioxidant compounds.

## Figures and Tables

**Table 1:** IC_50_ value of ABTS radical scavenging assay of heated MAAs.

Compounds	pH			
	5.8	6.6	7.4	8.0
	(µM)			
Palythine *	>72.0	>72.0	23.4	12.0
Heated palythine	11.4	8.7	8.7	6.7
Porphyra-334 *	>72.0	>72.0	27.5	20.8
Heated porphyra-334	12.8	13.4	12.4	6.3
Ascorbic acid *	19.1	19.4	12.4	8.9

Values are expressed as means of three independent experiments. * Data from reference [14].

**Table 2:** NMR data of palythine and heated palythine in D_2_O.

Position	Palythine *		Heated Palythine	
	*δ*^1^H (ppm)	*δ*^13^C (ppm)	*δ*^1^H (ppm)	*δ*^13^C (ppm)
1	-	161.5	-	165.9
2	-	125.9	-	127
3	-	158.7	-	160.9
4	2.60 (d)	33.5	4.58 (s) or 4.66 (s)	144.4
	2.75 (d)			
5	-	71.2	-	141.2
6	2.58 (d)	35.7	4.58 (s) or 4.66 (s)	29
	2.86 (d)			
7	3.48 (s)	67.3	3.81 (s)	65.8
8	3.56 (s)	58.9	3.87 (s)	63.5
9	3.95 (s)	47	4.13 (s)	47.4
10	-	174.6	-	178.2

Numbers of positions correspond to Figure 4. * Data from reference [3]. -, Not detected.

## Data Availability

Not applicable.

## Supplementary Materials

The following supporting information can be downloaded at: https://www.mdpi.com/article/10.3390/md20030184/s1, Figure S1: ESI-MS of heated palythine, Figure S2: ^1^H-NMR of heated palythine, Figure S3: ^13^C-NMR of heated palythine.
