# Supplementary material for: Characterization of Antioxidant Activity of Heated Mycosporine-like Amino Acids from Red Alga Dulse Palmaria palmata in Japan
Source: Mar Drugs. 2022 Mar 1;20(3):184. doi: 10.3390/md20030184 (PMC8954034; doi:10.3390/md20030184)
Supplement: Supplementary file 1 [file marinedrugs-20-00184-s001.zip › marinedrugs-1614202-supplementary.pdf]

Supplementary Materials

Characterization of Antioxidant Activity of Heated Mycosporine-Like Amino Acids from Red Alga Dulse *Palmaria palmata* in Japan

Yuki Nishida [1], Wataru Saburi [2], Yoshikatsu Miyabe [1,3], Hideki Kishimura [4,*] and Yuya Kumagai [4,*]

[1] Marine Chemical Resource Development, Graduate School of Fisheries Sciences, Hokkaido University, Hakodate 041-8611, Hokkaido, Japan; karakuchi@eis.hokudai.ac.jp (Y.N.)
[2] Fundamental AgriScience Research, Research Faculty of Agriculture, Hokkaido University, Kita 9 Nishi 9, Kita-ku, Sapporo, 060-8589, Japan; saburi@chem.agr.hokudai.ac.jp (W.S.)
[3] Food Research Institute, Aomori Prefectural Industrial Technology Research Center, 2-10 Chikkogai, Hachinohe-shi 031-0831, Aomori-ken, Japan; yoshikatsu_miyabe@aomori-itc.or.jp (Y.M.)
[4] Marine Chemical Resource Development, Faculty of Fisheries Sciences, Hokkaido University, Hakodate 041-8611, Hokkaido, Japan; i-dulse@fish.hokudai.ac.jp (H.K.); yuyakumagai@fish.hokudai.ac.jp (Y.K.)
* Correspondence: i-dulse@fish.hokudai.ac.jp (H.K.); yuyakumagai@fish.hokudai.ac.jp (Y.K.)

(a)

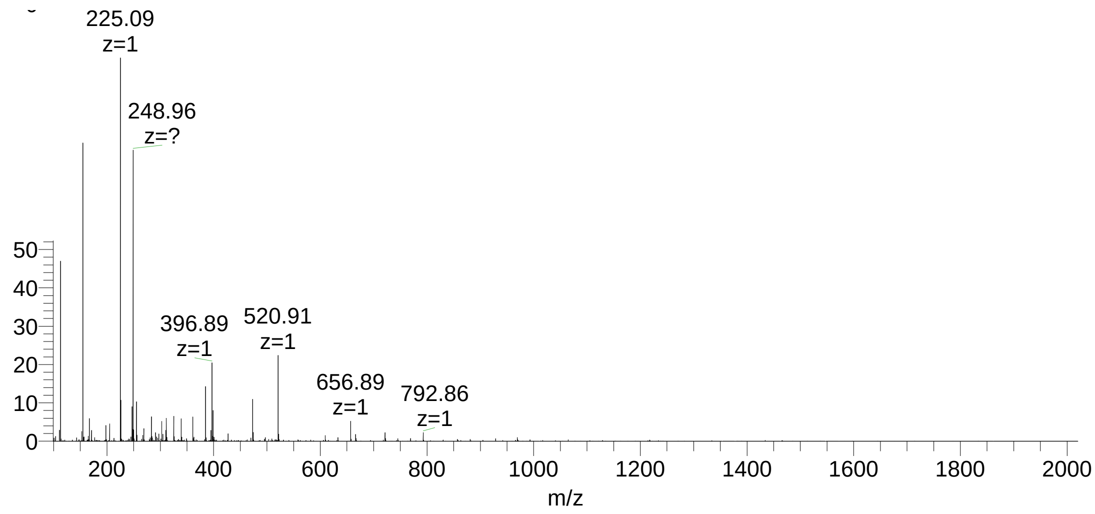

(b)

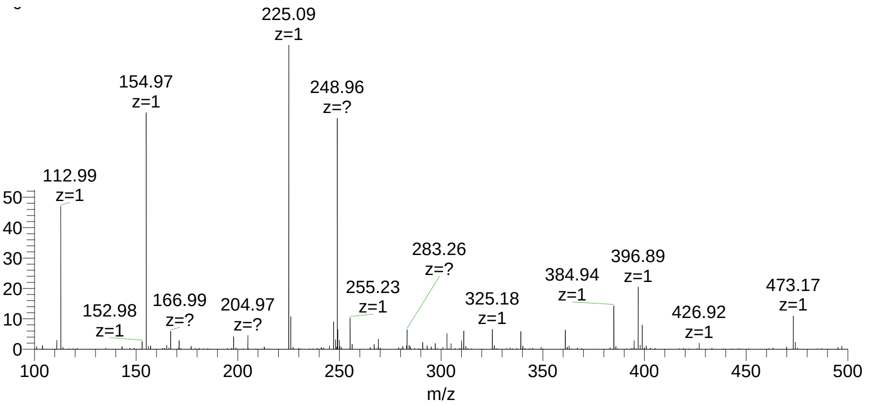

Figure S1. ESI-MS of heated palythine. (a) Whole range of 100-2,000 *m/z*, (b) zoom up range of 100-500 *m/z*.

(a)

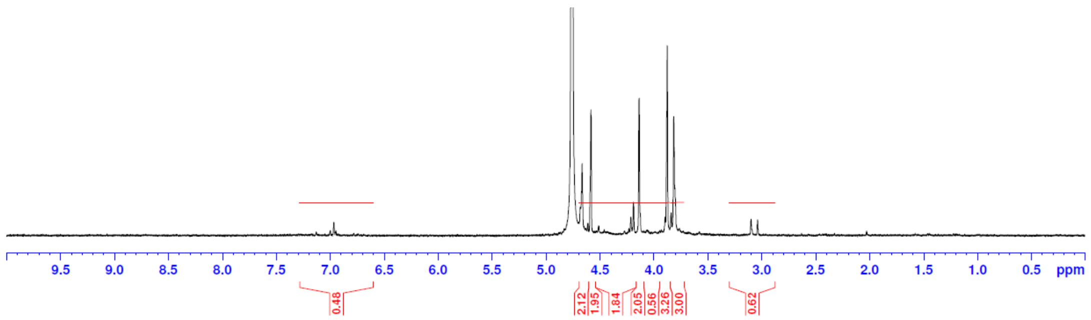

(b)

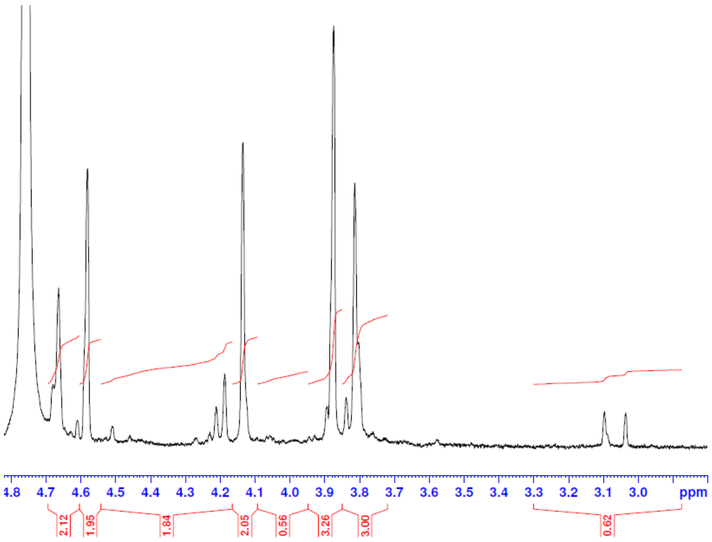

Figure S2. ^{1}H-NMR of heated palythine. (a) Whole, (b) zoom up.

(a)

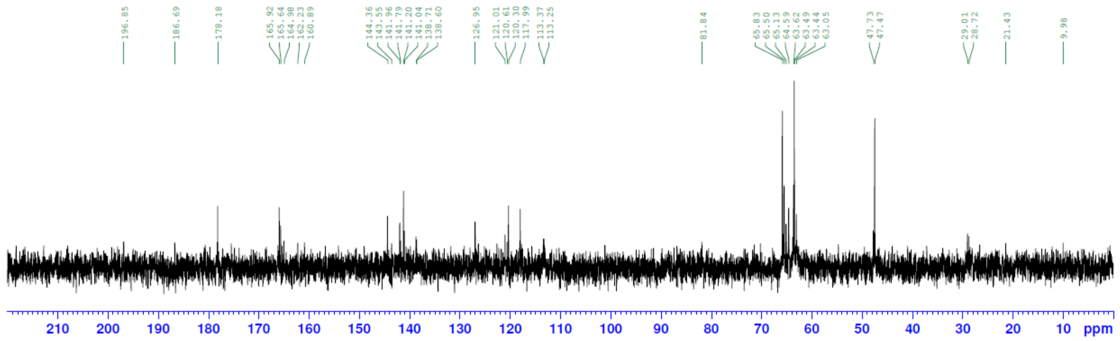
196.85
186.69
178.18
165.92 165.64 164.98 162.23 160.89
144.36 143.55 141.96 141.79 141.20 141.04 138.71 138.60
126.95
121.01 120.61 120.30 117.99
113.37 113.25
81.84
65.83 65.50 65.13 64.59 63.62 63.49 63.44 63.05
47.73 47.47
29.01 28.72
21.43
9.98
210 200 190 180 170 160 150 140 130 120 110 100 90 80 70 60 50 40 30 20 10 ppm

(b)

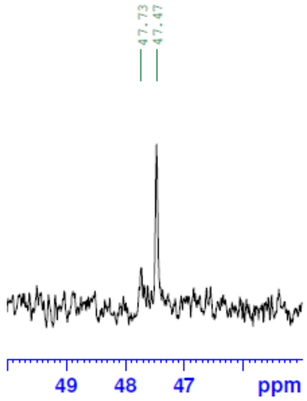
47.73
47.47
49 48 47 ppm

(c)

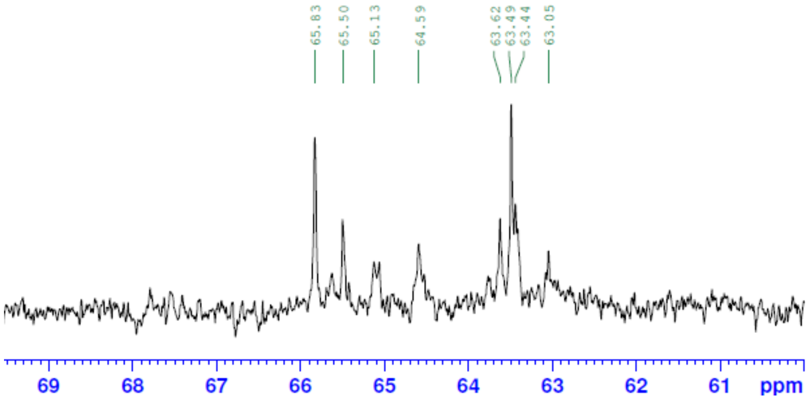
65.83
65.50
65.13
64.59
63.62 63.49 63.44
63.05
69 68 67 66 65 64 63 62 61 ppm

(d)

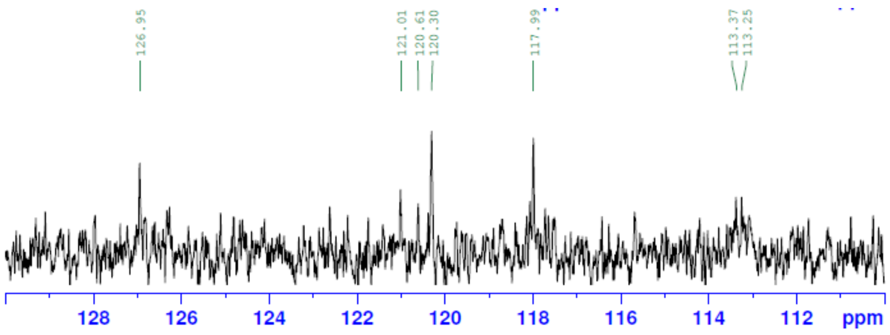

(e)

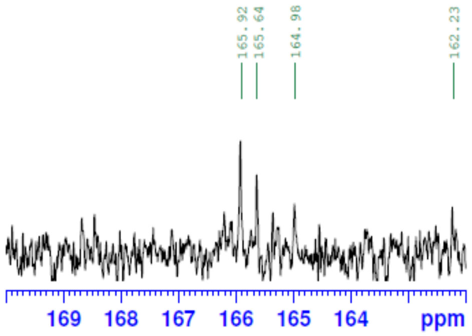

Figure S3. ^{13}C-NMR of heated palythine. (a) Whole, (b) zoom up of 47-49 ppm, (c) zoom up of 61-69 ppm, (d) zoom up of 112-128 ppm, (e) zoom up of 164-169 ppm.
